# Advanced glycation end products consumption and the decline of functional capacity in patients with Parkinson's disease: Cross-sectional study

**DOI:** 10.1016/j.clinsp.2023.100320

**Published:** 2024-01-31

**Authors:** Jenifer Kristina Alves de Almeida Biase, Guilherme Carlos Brech, Natália Mariana Silva Luna, Rodrigo Tallada Iborra, Jose Maria Soares-Junior, Edmund Chada Baracat, Júlia Maria D'Andrea Greve, Angélica Castilho Alonso, Adriana Machado-Lima

**Affiliations:** a Graduate Program in Aging Sciences, Universidade São Judas Tadeu (USJT), São Paulo, SP, Brazil; b Laboratory Study of Movement, Instituto de Ortopedia e Traumatologia do Hospital das Clínicas (IOT-HC) da Faculdade de Medicina da Universidade de São Paulo (FMUSP), São Paulo, SP, Brazil; c Disciplina de Ginecologia, Departamento de Obstetrícia e Ginecologia, Hospital das Clínicas, Faculdade de Medicina da Universidade de São Paulo (FMUSP), São Paulo, SP, Brazil

**Keywords:** Advanced glycation end-products, Parkinson's disease, Aging, Functional capacity

## Abstract

**Introduction:**

Advanced Glycation End-Products (AGEs) are a diverse group of highly reactive molecules that play a vital role in the development of neurodegenerative disorders, such as Parkinson's Disease (PD), leading to a decline in functional and cognitive capacity. The objective of this study was to assess the intake and quantification of AGEs in individuals with PD and to correlate them with their functional and cognitive abilities.

**Methods:**

This was a cross-sectional study involving 20 PD patients and 20 non-PD individuals as the Control group (C). The autofluorescence reader was used to evaluate skin AGEs, while food recall was used to quantify AGEs consumed for three different days. The Montreal Cognitive Assessment, Short Physical Performance Battery, and handgrip tests were used. PD patients demonstrated greater impairment in functional capacity compared to the control group.

**Results:**

Dominant Handgrip (p = 0.02) and motor performance, in the sit and stand test (p = 0.01) and Short Physical Performance Battery (SPPB) (p = 0.01) were inferior in PD patients than the control group. Although PD patients tended to consume less AGEs than the control group, AGE intake was negatively correlated with handgrip strength in individuals with PD (*r* = -0.59; p < 0.05).

**Conclusion:**

PD patients had lower strength and functional capacity, suggesting that the effects of AGEs might be exacerbated during chronic diseases like Parkinson's.

## Highlights

• Effects of AGEs in Parkinson's disease patients.
• Recommendation healthy diet with a low AGE consumption.
• Parkinson's disease patients’ lower strength and worsened functional capacity.

## Introduction

Advanced Glycation End-Products (AGEs) are a heterogeneous group of highly reactive molecules that are formed by the reaction of glycation or Maillard reaction [1,2]. AGE has been described as playing an important role in the development of neurodegenerative diseases [3]. The glycation appears to trigger abnormal deposition of modified proteins, which induces local oxidative stress, resulting in an inflammatory response. Consequently, cells become dysfunctional and could die, giving rise to pathological and clinical aspects of neurodegenerative diseases, this could be a consequence of AGE accumulation on the central and peripheral nervous system [4].

Parkinson's Disease (PD) is the second most common neurodegenerative disease [1] and its incidence increases gradually in older adults.^5^ Cognitive impairment is an important non-motor characteristic of PD, and dementia can occur in more than 80 % of PD patients after 20 years of illness.^6^ Furthermore, Parkinson's disease is characterized by a gradual deterioration of dopaminergic innervation in different areas of the brain. This leads to motor symptoms like rigidity, gait and balance issues, bradykinesia, and tremors, as well as non-motor symptoms such as cognitive, autonomic, and sensory impairments [7].

Glycation seems to increase the formation of aggregated proteins during PD: the α-synuclein, has 15 lysine residues and is more susceptible to glycation, contributing to neuronal cell dysfunction [8]. In other studies, the presence of AGEs in Lewy bodies has already been evidenced [9,10] and in the cerebral cortex of PD patients [11]. More recently, Chegao et al. [12] demonstrated that the glycation exacerbated behavioral phenotype might result from the dysregulation of multiple neurotransmission pathways in the midbrain, wichi could accelerate PD-like sensorimotor and cognitive alterations and suggest that the increase of glutamatergic signaling may underlie these events.

During the aging process, a decline in functional capacity, a reduction in walking speed, unstable balance and loss of muscle strength [13]. Increased AGE formation in relevant brain regions related to motor function may have important effects on the biomechanical properties of peripheral musculoskeletal and neurological tissues, which may have a significant impact on motor function [8]. A study from Germany shows that PD patients have higher plasma levels of one type of AGE ‒ Carboxymethyl Lysine (CML), compared to healthy individuals [14].

Diet has been associated as a risk factor for the development of PD and also as a protective factor for the worsening of the disease. For example, a diet that includes a high intake of fruits, vegetables, legumes and fish is inversely associated with the risk for developing PD [15]. A high AGEs diet, on the other hand, has been associated with an increased incidence of neurodegenerative diseases, such as Alzheimer's disease [16].

AGEs from food can be absorbed and transported into the circulation by albumin and lipoproteins [17]. Although oral bioavailability is considered low (-10 %), studies in healthy humans have shown that dietary AGEs correlate directly with circulating AGEs, such as CML and Methylglyoxal (MG), as well as with markers of oxidative stress [17]. Furthermore, restricting dietary AGEs in individuals in addition to reducing markers of oxidative stress and inflammation in healthy individuals, it also has the same effect in individuals with diabetes or kidney disease [18].

Many studies have shown the relationship between the formation of early glycation products such as glycated hemoglobin and PD. In a prospective and observational study realized in Hamburg, Germany, with PD patients, diabetes and high glycated hemoglobin were associated with increased neuroaxonal damage and cognitive impairment [19]. In study study other cross-sectional studies, from Korea, higher glycated hemoglobin levels significantly correlated with lower MoCA scores in PD patients [20]. Glycated hemoglobin was independently associated with disease severity in PD patients from Beijing, China [21]. However, there is not a full understanding yet about what are the effects of the formation and consumption of AGEs and their relationship with the incidence and progression of Parkinson's disease in older adults. In this sense, our research aims to estimate the consumption and quantification of advanced glycation products in patients with Parkinson's disease and to relate to their functional and cognitive capacity in older adults.

## Materials and methods

### Study design and local

This is a cross-sectional study, developed at the Universidade São Judas Tadeu in partnership with the Laboratory of the Study of Movement of the Institute of Orthopedics and Traumatology (IOT) of Hospital das Clínicas (HC) of the Faculty of Medicine of the University of São Paulo (FMUSP). Approved by the Ethics Committee of the USJT nº 79991417900000089. All participants signed the free and informed consent form.

### Participants

This is a conventional sample, composed of 20 Parkinson's Disease (PD) patients, 10 females and 10 males, and 20 individuals, with the same gender distribution and paired by age, not suffering from PD to compose the control group. Included individuals older than 55 years. Individuals who were unable to perform the Short Physical Performance Battery (SPPB) were not included in the study, and there was no restriction on the level of schooling. In addition, to motor characterization of the sample was applied the third part (motor) of the Unified Parkinson's Disease Rating Scale (UPDRS) evaluates 18 motor examinations, including speech, facies, resting tremor, intentional tremor, rigidity, rapid finger movements, rapid hand movements, alternating movements, leg movements, getting up from a chair, posture, stability of posture, starting walking, and bradykinesia. The scoring system assigns points based on the degree of involvement in each examination, with 0 points indicating no involvement, 1 point indicating detectable disorders, 2 points indicating moderate disorders, 3 points indicating considerable disorders, and 4 points indicating no function or severe disorders. The total score, is the sum of all these items.

### Assessment

For anthropometric evaluation, were measured body mass, height, and Abdominal Circumference (AC). The Body Mass Index (BMI) was used considering weight per height ratio (kg/m^2^). The AGE intake was analyzed based on 3-day food records, which allowed the estimation of consumed food and beverages. A nutritionist, trained in dietary recall, used a form with questions about the participants' food intake, such as the consumption of fried, grilled, boiled, and roasted foods during the week.

She examined the patient records that provided information on the sizes of food portions and the methods used for cooking. Using Microsoft Excel (Microsoft®), she estimated the content of AGEs from a database of about 560 different foods [22] and their corresponding AGE values (based on CML content). The results were expressed in AGE Equivalents (Eq) per day, with 1,000 kilounits equaling one AGE Eq, [22] The same estimative was performed previously in subjects with Diabetes Mellitus [2]. Also, it were asked about tobacco use from the participants.

The level of Skin Autofluorescence was determined using the AGE Reader device (DiagnOptics Technologies BV, Groningen, the Netherlands). This is a convenient, non-invasive tool that utilizes the fluorescent properties of specific AGEs to estimate the accumulation of AGEs in the skin [23]. The standard measuring site for skin AF is the right forearm, which was placed on the device during the assessment. The AGE Reader illuminates a skin surface of 4 cm^2^ with an excitation light source that has a peak excitation of 370 nm. A spectrometer then measures the emitted light (fluorescence in the 420‒600 nm wavelength) and the reflected excitation light (in the 300‒420 nm wavelength) from the skin. Skin AF is calculated by taking the ratio of the emitted light to reflected excitation light, multiplying it by 100, and expressing the result in arbitrary units [23]. Three consecutive measurements were performed, taking less than a minute. The mean skin AF value from these three measurements was then calculated and used in the subsequent analyses.

For cognitive evaluation of the groups, the Montreal Cognitive Assessment (MoCA) was used. MoCA consisted of both verbal and pencil/paper tasks, developed as a brief screening tool for mild cognitive impairment. The same access different cognitive domains: Attention and concentration, executive functions, memory, language, visuospatial skills, conceptualization, calculation, and orientation [24]. MoCA scores were adjusted for education by adding one point to subjects with 12 or fewer years of education.

The Short Physical Performance Battery (SPPB) was used to evaluate the balance, which is composed of three tests: a hierarchical assessment of standing balance, a short walk at the usual elderly pace (gait speed), and standing five times from a seated position in a chair (Sit to Stand test). In order to measure the Hand Grip Strength (HGS), the JAMAR® manual hydraulic dynamometer was used, respecting the protocol of the American Association of Hand Therapists (ASHT). The study participants were positioned in a seated posture with their back supported, hips and knees flexed at a 90-degree angle, feet in contact with the ground, and their elbows flexed at 90 degrees with the forearm and wrist in a neutral position. They were instructed to hold the dynamometer and squeeze it with the maximum force possible. The tests were conducted alternately between the dominant and non-dominant hand, with a one-minute interval between each measurement. Three measurements were taken for each hand, and the highest value was selected for analysis to represent the maximum strength capacity of the dominant and non-dominant hand. To evaluate muscle mass, the equation validated for the Brazilian population of Lee [25] was used to estimate appendix muscle mass where MMEA=0.2344×weight+7.80×height+6.6×sex−0.098×age+race/ethnicity−3.3. [25] In this way the values were adjusted by the squared height and the Total Muscle Mass Index (MMT/E2) was established.

### Statistical analysis

Statistical analyzes were performed using the Graph Pad Prism 9.0 and Statistical Package for Social Sciences (SPSS) [24]. The Shapiro-Wilk normality test was applied and followed by Student's *t*-test. It was considered significant p < 0.05. Data were expressed as mean ± standard deviation. Simple linear regression (forward mode) to investigate whether independent variables predict the cognitive and functional capacity of individuals with and without PD The regression model variables were included in the following order Model 1: General data (age, PD duration, comorbidities and medications); Model 2: General data (age, PD duration, comorbidities and medications) and nutritional (AGE); Model 3: General data (age, PD duration, comorbidities and medications), nutritional and anthropometric characteristics and Model 4: General data (age, PD duration, comorbidities and medications), nutritional and anthropometric characteristics and physical performance. Comorbidities were described as the participants reported such as dyslipidemia, hypertension, and diabetes. The participants also report the medications that were used to treat PD, dyslipidemia, hypertension, and diabetes.

## Results

The groups included 20 control subjects and 20 PD individuals, both with 10 women and 10 men with similar average age. Table 1 shows the descriptive analysis of the general data: anthropometry, cognitive and functional capacity, for control group and PD subjects group (PD group).

**Table 1:** Characterization of control subjects (Control) and Parkinson's Disease patients (PD).

	**PD (n = 20)**	**Control (n = 20)**	***t***	**p**	**95% CI**
**General data**					
Age (years)	70 ± 10.3	69 ± 6.1	0.66	0.52	-7.16; 3.65
Associated comorbidities	0.9 ± 1.0	0.8 ± 0.7	0.73	0.35	-0.67; 0.47
Family income (US Dolar)	1512 ± 773	1460 ± 843	0.21	0.84	-571; 464
Medicines for continuous use	3.7 ± 2.4	1.9 ± 1.7	2.58	0.01^a^	-3036; -0.36
UPDRS	9.15 ± 5.8	‒			
**Anthropometry**					
BMI (kg/m^2^)	26.4 ± 2.9	27.1 ± 3.7	1.23	0.23	-3.41; 0.83
AC (cm)	98.8 ± 10.2	91.1 ± 10.8	2.31	0.03	-14.40; -0.95
**Cognitive ability**					
MoCA	22.5 ± 4.2	24.8 ± 3.1	1.63	0.11	-0.47; 4.27
**Functional capacity**					
Handgrip test DS (Kg/f)	18.8 ± 6.9	27. 1 ± 12.9	2.52	0.02^a^	1.64; 14.90
Handgrip test NDS (Kg/f)	19.4 ± 9.4	24.3 ± 10.5	1.54	0.13	-1.53; 11.21
Gait speed (m/s)	7.2 ± 4.8	6.1 ± 2.1	0.96	0.34	-3.51; 1.25
Sit to Stand test (s)	15.1 ± 3.0	11.7 ± 3.7	3.19	0.01^a^	-5.55; -1.24
SPPB	8.5 ± 2.2	10 ± 1.5	2582	0.01^a^	0.34; 2.76
**AGE Formation**					
AF (AU)	2.64 ± 0.61	3.06 ± 0.52	2.18	0.04	0.03; 0.80
Rec 24hrs (kU/d)	17028 ± 11392	24561 ± 15187	1.78	0.08	-1060; 16127
Rec 48hrs (kU/d)	18182 ± 11439	24981 ± 13077	1.75	0.09	-1066; 14664
Rec habitual (kU/d)	19722 ± 8072	24091 ± 15046	1.14	0.26	-3361; 3818
Average Consumption (kU/d)	18311 ± 6458	24544 ± 13384	1.88	0.07	-494; 12961
**AGE consumption from food (kU/d)**					
Grilled (days of the week)	1.450 ± 1.91	2.50 ± 19.33	2.07	0.05	0.02; 2.08
Frying (days of the week)	2.0 ± 1.92	0.65 ± 0.88	2.86	0.01^a^	-2.31; -0.40
Roast (days of the week)	1.60 ± 1.10	1.05 ± 0.99	1.66	0.11	-1.22; 0.12
Cooked (days of the week)	4.25 ± 2.29	5.00 ± 1.97	1.11	0.27	-0.62; 2.12

PD, Parkinson's Disease; AGE, Advanced Glycated Endproduct; UPDRS, Unified Parkinson Disease Rating Scale (UPDRS); AF, Autofluorescence of the skin; Rec, Reminder; kU/d, kU/day; UA, Arbitrary Units; BMI, Body Mass Index; AC, Abdominal Circumference; MoCA, Montreal Cognitive Assessment; SPPB, Short Physical Perfomance Battery; DS, Dominant Side; NDS, Non-Dominant Side; 95 % CI, 95 % Confidence Interval; s, seconds; m/s, meter per second; Kg/f, Kilo by force.

Student's *t*-test. Data were expressed as mean ± standard deviation.

a Level of significance adopted (p < 0.05).

No difference in cognitive ability was observed between the groups with and without PD assessed by the MoCA, this may be related to the fact that the 2 groups presented similar schooling, as this variable interferes in the final results of MoCa. In addition, when the results were classified according to the instrument scores, both groups presented cognitive decline. To estimate PD's motor function, we have used the UPDRS. PD showed the mean of total score characterized by active individuals who were able to walk.

Participants with PD have greater impairments regarding their functional capacity when compared to the control group. It was observed that the dominant side hand grip values were lower in individuals with PD. In addition, decreasing muscular strength and motor performance (Timed-up-and-go test) parameter declining were observed, and was evaluated by the SPPB test in PD patients. No difference was observed in the non-dominant side hand grip values between the control group and the Parkinson's group.

The individuals from the control group had a higher AGE presence in the skin, measured by skin Autofluorescence (AF), as compared to the participants in the PD group (Table 1). All participants were questioned about tobacco use, another important exogenous source of AGE, as no participant from this study had smoked. The control group had a tendency (p = 0.07) to consume more AGE when compared to the PD group (Table 1).

**Figure 1:**
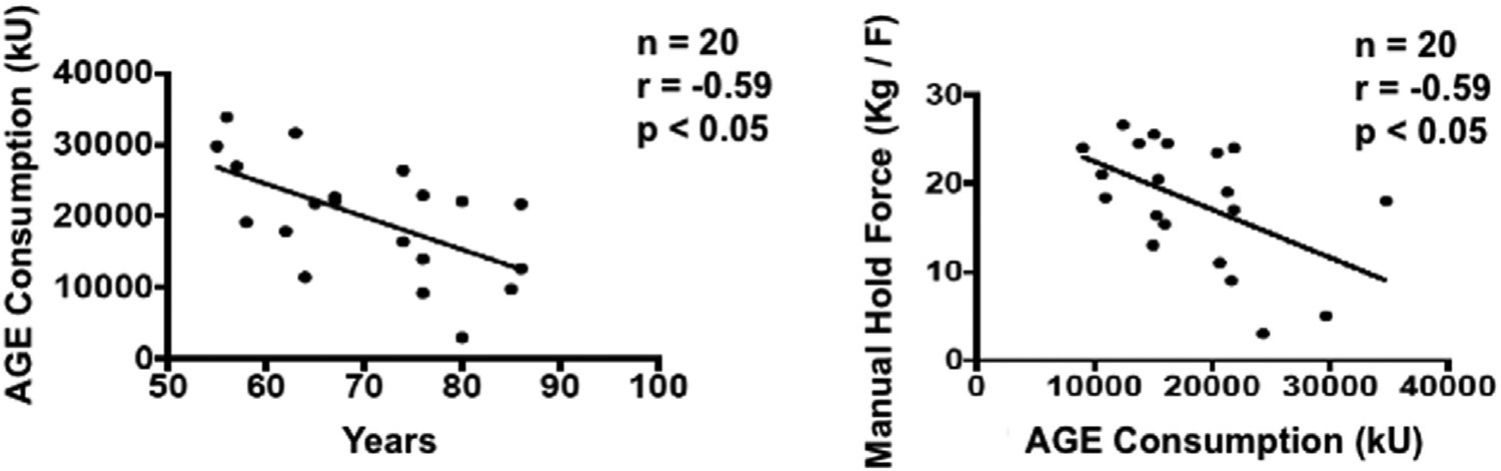
Pearson correlation between the variables Hand Grip strength (Kg/F) versus AGE Consumption (kU) panel A and AGE Consumption (kU) versus age (years), based on the average consumption of three distinct days (24 hours; 48 hours and usual) of the group of individuals with DP.

We observed a negative correlation between AGE consumption and age in the PD group (Fig. 1), which means that the older the PD patient, the lower their AGE consumption. The AGE consumption correlated negatively with the hand grip strength (Kg/F) in individuals with PD, therefore, the higher the AGE consumption, the lower the strength (Fig. 1). No correlation was observed in AGEs with the handgrip strength for Control (p = 0.84 e *r* = -0.04785).

Table 2 shows the multiple linear regression model for cognitive analysis for Control (n = 20) and PD (n = 20) groups. In Model 1, the independent variables age, comorbidities and medications explained 8 % in the control group and 7 % in the group with PD, of the MoCA value. In Model 2, the variables preview plus the formation consumption of AGE explained 12 % in the control group and 11 % in the PD group of the cognitive test. Model 3, composed by variables from Model 1 and 2, plus BMI and WC, explains 12 % of MoCA in the control group and 28 % in the group with PD. On the other hand, Model 4, combining the variables of Model 1, 2 and 3 with motor variables such as non-dominant and dominant side handgrip test, gait speed, sitting and rising together explained 17 % in the control group and 40 % in the group with PD, in the cognitive test.

**Table 2:** Model of cognitive ability in individuals of the control group and with Parkinson's disease.

**Control group**	**Model 1**		**Model 2**		**Model 3**		**Model 4**	
**Variables**	**β**	**EP**	**β**	**EP**	**β**	**EP**	**β**	**EP**
General data								
Age (years)	-0.10	0.08	-0.11	0.08	-0.12	0.09	-0.12	0.11
Comorbidities	-0.23	0.31	-0.12	0.33	-0.10	0.34	-0.17	0.36
Medicines	-0.10	0.08	-0.11	0.08	-0.12	0.09	-0.12	0.11
AGE								
AGE consumption (kU/d)			0.01	0.01	0.01	0.01	0.01	0.01
AF (AU)			0.93	0.88	0.97	0.92	0.73	0.10
Anthropometry								
BMI (kg/m^2^)					-0.10	0.42	-0.25	0.47
AC (cm)					0.01	0.12	0.06	0.13
Motor Domain								
Hand grip test NDS (kg/f)							-0.11	0.15
Hand grip test DS (kg/f)							0.02	0.14
Gait speed (m/s)							0.04	0.21
Sit to stand (s)							-0.15	0.23
R squared	**0.08**		**0.12**		**0.12**		**0.17**	
Parkinson disease group								
General data								
Age (years)	-0.12	0.11	-0.12	0.12	-0.09	0.13	0.22	0.22
Disease duration (years)	-0.07	0.34	-0.11	0.37	-0.14	0.36	0.45	0.45
Comorbidities	0.22	1.17	0.18	1.31	0.25	1.4	2.76	2.76
Medicines	0.23	0.51	0.09	0.65	-0.07	0.65	0.79	0.79
AGE								
AGE consumption (kU/d)			0.01	0.01	0.01	0.01	0.01	0.01
AF (AU)			1.12	1.68	1.13	1.68	2.57	2.57
Anthropometry								
BMI (kg/m^2^)					0.25	0.69	1.30	1.30
AC (cm)					0.12	0.18	0.30	0.30
Motor Domain								
Hand grip test NDS (kg/f)							0.35	0.35
Hand grip test DS (kg/f)							0.55	0.55
Gait speed (m/s)							0.35	0.52
Sit to stand (s)							0.45	0.89
R squared	**0.07**		**0.11**		**0.28**		**0.40**	

PD, Parkinson's Disease; β, Beta; EP, Standard Error; MoCA, Montreal Cognitive Assessment; AGE, Advanced Glycated Endproduct; AF, Autofluorescence of the skin; kU/d, kU/day; UA, Arbitrary Units; BMI, Body Mass Index; AC, Abdominal Circumference; NDS, Non-Dominant Side; DS, Dominant Side; s, seconds; m/s, meter per second; Kg/f, kilo by force; kg/m^2^, Kilogram per square meter; cm, centimeter.

In Table 3 is indicated the multiple linear regression model for physical performance, performed analysis for individuals from control group (n = 20) and individuals from the PD group (n = 20). In Model 1, the variables of the SPPB value explained 12 % in the control group and 33 % in the group with PD. In Model 2, the variables mentioned above plus the consumption of formation of AGE explained 23 % of the physical performance in the control group and 47 % in the group with PD. Model 3 was composed of variables from Model 1 and 2, plus BMI, WC and total muscle mass calculated by the validated equation for the Brazilian population of Lee, which explains 47 % of the SPPB in the control group and 67 % in the group with PD. Model 4, combining the variables of Model 1, 2 and 3 with motor variables such as handgrip test non-dominant and dominant side, together explained 53 % of physical performance in the control group and 68 % in the group with PD.

**Table 3:** Physical performance model (SPPB) in Control and Parkinson's subjects.

**Group control**	**Model 1**		**Model 2**		**Model 3**		**Model 4**	
**Variables**	**β**	**EP**	**β**	**EP**	**β**	**EP**	**β**	**EP**
General data								
Age (years)	-0.02	0.07	-0.04	0.07	-0.07	0.07	-0.07	0.09
Comorbidities	-0.28	0.23	-0.16	0.25	0.00	0.29	-0.02	0.31
Medicines	-0.02	0.07	-0.04	0.07	-0.07	0.07	-0.07	0.09
AGE								
AGE consumption (kU/d)			0.01	0.01	0.01	0.01	0.01	0.01
AF (AU)			-0.29	0.55	0.09	0.57	0.10	0.60
Anthropometry								
BMI (kg/m^2^)					0.19	0.35	0.11	0.39
AC (cm)					-0.12	0.10	-0.11	0.11
Total Muscle Mass (kg)					-0.36	0.51	-0.06	0.65
Motor Domain								
Hand grip test NDS (kg/f)							0.10	0.10
Hand grip test DS (kg/f)							-0.06	0.08
*r* square	**0.12**		**0.23**		**0.47**		**0.53**	
**Parkinson disease group**								
General data								
Age (years)	-0.13	0.05	-0.13	0.05	-0.184	0.051	-0.17	0.06
Disease duration (years)	-0.14	0.15	-0.09	0.15	-0.062	0.140	-0.06	0.15
Comorbidities	-0.20	0.53	-0.12	0.54	-0.616	0.538	-0.59	0.59
Medicines	0.16	0.23	0.28	0.27	0.321	0.253	0.33	0.28
AGE								
AGE consumption (kU/d)			0.01	0.01	0.01	0.01	0.01	0.01
AF (AU)			-1.22	0.70	-1.66	0.66	-1.60	0.78
Anthropometry								
BMI (kg/m^2^)					-0.41	0.28	-0.41	0.32
AC (cm)					0.15	0.07	0.14	0.08
Total Muscle Mass (kg)					-0.52	0.41	-0.36	0.53
**Motor Domain**								
Hand grip test NDS (kg/f)							0.08	0.14
Hand grip test DS (kg/f)							-0.09	0.20
R squared	**0.33**		**0.47**		**0.67**		**0.68**	

PD, Parkinson's disease; β, Beta; EP, Standard Error; MoCA, Montreal Cognitive Assessment; AGE, Advanced Glycated Endproduct; AF, Autofluorescence of the skin; kU/d, kU/day; UA, Arbitrary Units; BMI, Body Mass Index; AC, Abdominal Circumference; NDS, Non-Dominant Side; DS, Dominant Side; s, seconds; m/s, meter per second; Kg/f, kilo by force; kg/m^2^, Kilogram per square meter; cm, centimeter.

## Discussion

In this study, we evaluated the consumption and quantified the presence of skin Advanced Glycation End-Products (AGEs) in older adults control subjects and patients with Parkinson's Disease (PD) and their possible relationships with functional and cognitive capacity during the aging process. We found a positive correlation between AGE consumption and age with PD patients and a negative correlation between hand grip strength and AGE consumption, this means that PD patients who consumed more AGE presented lower strength.

No statistical difference was found in the cognitive ability, evaluated by the MoCA test among the participants. Regarding studies involving patients with PD, one study showed that patients with PD had lower cognitive performance than control groups without PD in several domains of MoCA [26].

In our study, cognitive ability did not correlate with skin AF and AGEs consumption. Frimat et al. [6] state that the impact of a diet with a high AGE content on cognitive function also remains ambiguous. In contrast, a study by Beeri et al. [9] demonstrated a decline in the Mini Mental State Examination (MMSE) performance in individuals with higher levels of serum methylglyoxal. In accordance with Beeri et al. [9], Poletti et al. [26] has also reported an important role of methylglyoxal on cognitive and neurodegenerative decline associated with the elderly [26].

When we performed linear regression models, on Table 2 we observed that age, time of PD, associated comorbidities, and use of medicines were responsible for 7 % of the cognitive ability in the control group and 8 % of PD patients. These percentages of both groups can be explained by their very similar characteristics, since there was no difference, except in the use of medication, where the PD group uses a greater number.

These variables added to the AGE consumption and skin AF quantification explain 12 % of the cognitive capacity in the control and 11 % in the PD group. The possible explanations for changes in cognitive ability are more evident in the Model 3 when the anthropometric variables were added to the model: explaining 12 % to the control and 28 % PD. Although, in both groups, participants were overweight, according to the BMI, the PD group had a significantly higher visceral fat, evaluated by AC, indicating that the subclinical conditions of the disease may contribute to an imbalance of the body composition, causing excessive adiposity in the abdominal region and reducing lean body mass [27]. The cognition impairment in PD described in the literature [26] could be related to the individual nutritional status. The mesial temporal cortex, which is involved in memory and controls food intake, is affected by dementia. These changes could affect the region involving the serotonin, dopamine and adrenaline production, which are involved in regulating the eating behavior and over time could compromise the nutritional status and the body composition of the individuals [28,29].

Finally, in the Model 4, where all the previous variables were added to the physical performance, explaining 17 % of the cognitive capacity in the control group 40 % and in the PD group. Evidencing the relationship between cognitive and motor skills in PD [25].

When we assessed the participants functional capacity, we observed a pronounced physical impairment in the PD group when compared to the control group, assessed by the SPPB test. It has been known that the gradual loss of muscle strength causes significant motor sequels in PD patients and can promote serious functional limitations. Assessing muscle strength is critical for evaluating the functional capacity of individuals and is widely used in clinical practice to estimate clinical outcomes over time and to predict prognosis [30].

In the linear regression Model 1 for physical performance, the variables explain 33 % of the physical capacity of PD patients and 12 % of the control group (Table 3). Aging is related to the decrease of activities and physical exercises,^31^ and associated with this, chronic diseases such as PD worsen this condition [32,33]. The treatment for PD symptoms is almost inevitable and represents a complex medication regimen [34].

When we associated the AGE consumption and skin AF quantification in linear regression (Table 3), these percentages increased to 47 % in the PD group and 23 % in the control group. Although the participants with PD consumed a lower amount of AGE when compared to the control group, this consumption is higher than is recommended by some authors [8,35] and therefore, it can exert some damage to the progression of Parkinson's disease. Furthermore, there is evidence that increased skin autofluorescence is related to neuropathies [36].

In our study, we found a negative correlation between hand grip strength and AGE consumption in the PD group, which means that PD individuals who consumed more AGE had lower strength. These results corroborate the hypothesis that AGE can pronounce the muscle atrophy in Parkinson's Disease. On the other hand, in recent years, there has been an increase of studies on the presence of peripheral neuropathy in Parkinson's disease [7]. It is suggested that peripheral neuropathy causes postural balance problems and muscle weakness. In our results we cannot rule out the influence of peripheral neuropathy on the Hand Grip strength results of the PD patients. Even the correlation between higher AGE consumption and lower hand grip strength may indicate a combined action between peripheral neuropathy and AGE consumption in individuals with PD, since the relationship between peripheral neuropathy and circulating AGEs is already well established in diabetes mellitus.^34^ More studies looking forward to evaluating the influence of AGEs on neuropathy in Parkinson's disease are necessary to elucidate this supposition.

When the anthropometric variables (BMI and AC) were inserted to Model 3, it explained 47 % of the physical performance in the control group and 67 % in PD; suggesting that the adiposity increased in the abdominal region may lead to worsening the physical capacity in PD patients [9].

Finally, when we add the hand grip values to the other variables, it explained 53 % of the functional capacity in the control group and 68 % in the PD group. Interesting to notice just a 1 % increase in the final model, which demonstrated the reduction of the hand grip strength in PD patients [37]. Therefore, it is important to notice that the decrease in strength in PD patients was to relate to an increase in AGE consumption.

PD patients selected for this study consumed approximately 18,311 kU/day of AGEs similar to other studies performed by our group: first study with DM1 patients with one type of microvascular complication could be diabetic kidney disease, retinopathy, peripheral neuropathy, or cardiovascular autonomic neuropathy in the same Brazilian region has shown similar ingestion of AGEs in diet (16,174 kU/day) [2] and other with healthy elderly subjects with no history of cardiovascular disease consume approximately 18,459±6766 AGE KU/day [38]. When PD patients were asked about the consumption of meat and animal products, some reported a restriction and others absence ingestion. This could explain the lower AGEs consumption in the PD group (18311±6458 KU/day) as compared to the control group (24544±13384 KU/day). PD patients usually consumed levodopa and they received an orientation to reduce ingestion of meat and animal products, because the amino acids consumption could interfere with the levodopa [34] and its inadequate administration could decrease the pharmacological effect of this substance due to drug-nutrient interaction [35].

Limitations of the study are related to the disease itself which is multifactorial with many interrelated predictor variables and perhaps, if we had biochemical data such as plasma AGEs and circulating RAGE, we could have more evidence to reinforce our findings. However, this work opens perspectives for new studies interrelating glycation, cognitive and physical capacity during the progression of Parkinson's disease.

## Conclusions

Parkinson's disease patients the individual showed decreased strength and subsequently reduced functional capacity, indicating that the impact of AGEs in the body may be heightened during chronic diseases like Parkinson's. Thus, it is suggested that the recommendation of a healthy diet with a low AGE consumption, may be an important way to reduce the progression of Parkinson's disease, however, further studies should be conducted to confirm these findings.

## Authors’ contributions

Jenifer Kristina Alves de Almeida Biase: Investigation and Writing-original draft and Supporting.

Guilherme Carlos Brech: Supporting Investigation and Writing-review & editing.

Natália Mariana Silva Luna: Writing-original draft.

Rodrigo Tallada Iborra: Investigation and Writing-original draft.

Jose Maria Soares-Junior: Supporting Investigation and Writing-review & editing.

Edmund Chada Baracat: Supporting Investigation and Writing-review & editing.

Júlia Maria D'Andrea Greve: Writing-review & editing.

Angélica Castilho Alonso: Formal analysis and Supporting Investigation and Writing-review & editing.

Adriana Machado-Lima: Supervisor and Writing-review & editing.

## Institutional review board statement

The study was conducted in accordance with the Declaration of Helsinki, and approved by the Ethics Committee of Ethical Committee for Human Research Protocols of the Sao Judas Tadeu University (protocol code number 79991417.9.0000.0089 date of approval November 2016

## Informed consent statement

Informed consent was obtained from all subjects involved in the study.

## Declaration of Competing Interest

The authors declare no conflicts of interest.
